# Diversity, functional classification and genotyping of SHV β-lactamases in Klebsiella pneumoniae

**DOI:** 10.1099/mgen.0.001294

**Published:** 2024-10-21

**Authors:** Kara K. Tsang, Margaret M. C. Lam, Ryan R. Wick, Kelly L. Wyres, Michael Bachman, Stephen Baker, Katherine Barry, Sylvain Brisse, Susana Campino, Alexandra Chiaverini, Daniela Maria Cirillo, Taane Clark, Jukka Corander, Marta Corbella, Alessandra Cornacchia, Aline Cuénod, Nicola D'Alterio, Federico Di Marco, Pilar Donado-Godoy, Adrian Egli, Refath Farzana, Edward J. Feil, Aasmund Fostervold, Claire L. Gorrie, Brekhna Hassan, Marit Andrea Klokkhammer Hetland, Le Nguyen Minh Hoa, Le Thi Hoi, Benjamin Howden, Odion O. Ikhimiukor, Adam W. J. Jenney, Håkon Kaspersen, Fahad Khokhar, Thongpan Leangapichart, Małgorzata Ligowska-Marzęta, Iren Høyland Löhr, Scott W. Long, Amy J. Mathers, Andrew G. McArthur, Geetha Nagaraj, Anderson O. Oaikhena, Iruka N. Okeke, João Perdigão, Hardik Parikh, My H. Pham, Francesco Pomilio, Niclas Raffelsberger, Andriniaina Rakotondrasoa, K. L. Ravi Kumar, Leah W. Roberts, Carla Rodrigues, Ørjan Samuelsen, Kirsty Sands, Davide Sassera, Helena Seth-Smith, Varun Shamanna, Norelle L. Sherry, Sonia Sia, Anton Spadar, Nicole Stoesser, Marianne Sunde, Arnfinn Sundsfjord, Pham Ngoc Thach, Nicholas R. Thomson, Harry A. Thorpe, M. Estée Torok, Van Dinh Trang, Nguyen Vu Trung, Jay Vornhagen, Timothy Walsh, Ben Warne, Hayley Wilson, Gerard D. Wright, Kathryn E. Holt, KlebNET-GSP AMR Genotype-Phenotype Group

**Affiliations:** 1 Department of Infection Biology, Faculty of Infectious and Tropical Diseases, London School of Hygiene & Tropical Medicine, London WC1E 7HT, UK; 2 Department of Infectious Diseases, School of Translational Medicine, Monash University, Melbourne, Victoria 3004, Australia; 3 Department of Microbiology and Immunology, The Peter Doherty Institute for Infection and Immunity, University of Melbourne, Melbourne, Victoria, Australia; 4 University of Michigan, Ann Arbor, USA; 5 University of Cambridge, Cambridge, UK; 6 University of Virginia, Charlottesville, USA; 7 Institut Pasteur, Université Paris Cité, Biodiversity and Epidemiology of Bacterial Pathogens, Paris, France; 8 Istituto Zooprofilattico Sperimentale dell'Abruzzo e del Molise "G. Caporale", Teramo, Italy; 9 Ospedale San Raffaele s.r.l. via olgettina, Milano, Italy; 10 University of Oslo, Oslo, Norway; 11 Microbiology and Virology Unit, Fondazione IRCCS Policlinico San Matteo, Pavia, Italy; 12 Institute of Medical Microbiology, University of Zurich, Zurich, Switzerland; 13 Centro de Investigación Tibaitatá de AGROSAVIA, Mosquera, Colombia; 14 Ineos-Oxford Institute for Antimicrobial Research, Department of Biology, University of Oxford, Oxford, UK; 15 The Milner Centre for Evolution, Department of Life Sciences, University of Bath, BA2 7AY, Bath, UK; 16 Department of Medical Microbiology, Stavanger University Hospital, Stavanger, Norway; 17 Cardiff University, Cardiff, Wales, UK; 18 National Hospital for Tropical Diseases, Hanoi, Vietnam; 19 Hanoi Medical University, Hanoi, Vietnam; 20 Department of Pharmaceutical Microbiology, University of Ibadan, Ibadan, Nigeria; 21 Norwegian Veterinary Institute, Ås, Norway; 22 Statens Serum Institut, Copenhagen, Denmark; 23 Houston Methodist, Weill Cornell Medical College, New York, USA; 24 Michael G. DeGroote Institute for Infectious Disease Research and Department of Biochemistry & Biomedical Sciences, McMaster University, Hamilton, Canada; 25 Central Research Laboratory, Kempegowda Institute of Medical Sciences, Bengaluru, India; 26 University of Lisbon, Lisbon, Portugal; 27 Wellcome Sanger Institute, Hinxton, UK; 28 Department of Microbiology and Infection Control, University Hospital of North Norway, Tromsø, Norway; 29 Institut Pasteur de Bangui, Bangui, Central African Republic; 30 Queensland University of Technology, Brisbane, Australia; 31 Norwegian National Advisory Unit on Detection of Antimicrobial Resistance, Department of Microbiology and Infection Control, University Hospital of North Norway, Tromsø, Norway; 32 Department of Pharmacy, Faculty of Health Sciences, UiT The Arctic University of Norway, Tromsø, Norway; 33 Department of Biology and Biotechnology, University of Pavia, Pavia, Italy; 34 Research Institute for Tropical Medicine, Department of Health, Manila, Philippines; 35 Nuffield Department of Medicine, University of Oxford, Oxford, UK; 36 Department of Medical Biology, Faculty of Health Sciences, UiT The Arctic University of Norway, Tromsø, Norway; 37 Indiana University School of Medicine, Indianapolis, USA; 38 PHG Foundation, University of Cambridge, Cambridge, UK

**Keywords:** AMR, β-lactamase, BLI resistance, extended-spectrum β-lactamase (ESBL), genotype, *K. pneumoniae*, prediction, SHV

## Abstract

Interpreting the phenotypes of *bla*_SHV_ alleles in *Klebsiella pneumoniae* genomes is complex. Whilst all strains are expected to carry a chromosomal copy conferring resistance to ampicillin, they may also carry mutations in chromosomal *bla*_SHV_ alleles or additional plasmid-borne *bla*_SHV_ alleles that have extended-spectrum β-lactamase (ESBL) activity and/or β-lactamase inhibitor (BLI) resistance activity. In addition, the role of individual mutations/a changes is not completely documented or understood. This has led to confusion in the literature and in antimicrobial resistance (AMR) gene databases [e.g. the National Center for Biotechnology Information (NCBI) Reference Gene Catalog and the β-lactamase database (BLDB)] over the specific functionality of individual sulfhydryl variable (SHV) protein variants. Therefore, the identification of ESBL-producing strains from *K. pneumoniae* genome data is complicated. Here, we reviewed the experimental evidence for the expansion of SHV enzyme function associated with specific aa substitutions. We then systematically assigned SHV alleles to functional classes (WT, ESBL and BLI resistant) based on the presence of these mutations. This resulted in the re-classification of 37 SHV alleles compared with the current assignments in the NCBI’s Reference Gene Catalog and/or BLDB (21 to WT, 12 to ESBL and 4 to BLI resistant). Phylogenetic and comparative genomic analyses support that (i) SHV-1 (encoded by *bla*_SHV-1_) is the ancestral chromosomal variant, (ii) ESBL- and BLI-resistant variants have evolved multiple times through parallel substitution mutations, (iii) ESBL variants are mostly mobilized to plasmids and (iv) BLI-resistant variants mostly result from mutations in chromosomal *bla*_SHV_. We used matched genome–phenotype data from the KlebNET-GSP AMR Genotype-Phenotype Group to identify 3999 *K*. *pneumoniae* isolates carrying one or more *bla*_SHV_ alleles but no other acquired β-lactamases to assess genotype–phenotype relationships for *bla*_SHV_. This collection includes human, animal and environmental isolates collected between 2001 and 2021 from 24 countries. Our analysis supports that mutations at Ambler sites 238 and 179 confer ESBL activity, whilst most omega-loop substitutions do not. Our data also provide support for the WT assignment of 67 protein variants, including 8 that were noted in public databases as ESBL. These eight variants were reclassified as WT because they lack ESBL-associated mutations, and our phenotype data support susceptibility to third-generation cephalosporins (SHV-27, SHV-38, SHV-40, SHV-41, SHV-42, SHV-65, SHV-164 and SHV-187). The approach and results outlined here have been implemented in Kleborate v2.4.1 (a software tool for genotyping *K. pneumoniae*), whereby known and novel *bla*_SHV_ alleles are classified based on causative mutations. Kleborate v2.4.1 was updated to include ten novel protein variants from the KlebNET-GSP dataset and all alleles in public databases as of November 2023. This study demonstrates the power of sharing AMR phenotypes alongside genome data to improve the understanding of resistance mechanisms.

**Impact Statement**

Since every *K. pneumoniae* genome has an intrinsic SHV β-lactamase and may also carry additional mobile forms, the correct interpretation of *bla*_SHV_ genes detected in genome data can be challenging and can lead to *K. pneumoniae* being misclassified as ESBL-producing. Here, we use matched *K. pneumoniae* genome and drug susceptibility data contributed from dozens of studies, together with a systematic literature review of experimental evidence, to improve our understanding of *bla*_SHV_ allele variation and mapping of genotype to phenotype. This study shows the value of coordinated data sharing, in this case via the KlebNET-GSP AMR Genotype-Phenotype Group, to improve our understanding of the evolutionary history and functionality of *bla*_SHV_ genes. The results are captured in an open-source AMR dictionary utilized by the Kleborate genotyping tool, which could easily be incorporated into or used to update other tools and AMR gene databases. This work is part of the wider efforts of the KlebNET-GSP group to develop and support a unified platform tailored for the analysis and interpretation of *K. pneumoniae* genomes by a wide range of stakeholders.

## Data Summary

*Bla*_SHV_ allele sequences and class assignments are distributed with Kleborate v2.4.1 (DOI: 10.5281/zenodo.10469001). The following supplementary tables are included in Supplementary Material 1. Table S1 provides a summary of *bla*_SHV_ alleles, including primary accessions, class-modifying mutations and supporting evidence for class assignments that differ from the NCBI’s Reference Gene Catalog or BLDB. The whole-genome sequence data are publicly available as reads and/or assemblies, and individual accessions are given in Table S2; corresponding genotypes and antibiotic susceptibility phenotypes and measurements are available in Tables S3 and S4, respectively; genomic location of SHVs is summarised in Table S5.

## Introduction

*Klebsiella pneumoniae* are typically resistant to ampicillin due to the production of a chromosomally encoded Ambler class A β-lactamase enzyme, sulfhydryl variable (SHV). Indeed, the European Committee on Antimicrobial Susceptibility Testing (EUCAST) terms this ‘expected resistance’ (formerly ‘intrinsic resistance’) and recommends against phenotypic testing of ampicillin resistance in *K. pneumoniae*, as a susceptible result is likely to be incorrect.

Whilst *bla*_SHV_ is a core chromosomal gene in *K. pneumoniae*, it has been mobilized out of the *K. pneumoniae* chromosome at least twice via IS*26* transposition [1], into multiple plasmid backbones [2] that, in turn, have spread between bacterial species. Chromosomal and plasmid forms of SHV have undergone allelic diversification to generate variants with differing functional activity, including extended-spectrum β-lactamases (ESBLs) conferring resistance to third-generation cephalosporins (3GCs) and alleles conferring resistance to β-lactamase inhibitors (BLIs).

The ESBL phenotype is facilitated through the overexpression of IS*26* [3] (e.g. SHV-2 and SHV-12). Consequently, *bla*_SHV_ genes identified in species other than *K. pneumoniae* are typically mobile and confer ESBL activity due to IS*26*, leading to a general conflation of SHV enzymes with ESBL. The existence of *bla*_SHV_ alleles with different activity profiles, including the potential for both chromosomal- and plasmid-encoded genes with differing functions in a single isolate, has created confusion and can lead to an incorrect interpretation of the phenotypic impact of the molecular detection of *bla*_SHV_ genes in *K. pneumoniae*.

It is not surprising that confusion exists around the interpretation of SHV variants, given the circuitous routes through which current understanding of the origins and evolution of the enzyme has emerged. First described in 1972 as a plasmid-encoded protein of *Escherichia coli* str. 453 conferring resistance to ampicillin [4], the enzyme was later given the name SHV-1 and was reported in multiple *Klebsiella*, *E. coli* and *Proteus mirabilis* isolates [5]. A 1979 study reported the *bla*_SHV-1_ gene as being chromosomally located in several *Klebsiella*, with a second plasmid-borne copy in one strain [6]. That study also demonstrated the transposition of the *bla*_SHV-1_ gene into different plasmid backbones and reported the detection of *bla*_SHV-1_ on naturally occurring plasmids of diverse types [6]. A naturally occurring plasmid-encoded variant, designated SHV-2 [7], displayed ESBL activity and conferred resistance to 3GCs, was reported in *K. pneumoniae*, *K. ozaenae* (now known as *K. pneumoniae* subsp. *ozaenae*) and *Serratia marcescens* in 1983 [8]. SHV-2 differs from SHV-1 by a single substitution (Gly to Ser) at Ambler codon 238 (aa 213 of the mature protein), which is sufficient to change its spectrum of activity [9]. *Bla*_SHV-2_ and *bla*_SHV-12_ are well known to be plasmid borne, following the transposition from the *K. pneumoniae* chromosome by IS*26* [1], and found outside *K. pneumoniae* [10,11]. By 1997, 12 protein variants of SHV had been reported, most of them ESBL and mostly in *K. pneumoniae* [2]. These were designated consecutive numbers (SHV-3, -4, etc.), with the exception of the ESBL variant SHV-2a, so named due to its similar kinetic properties to SHV-2, although it is not derived from SHV-2 [12].

Besides codon 238S, aa substitutions identified as conferring ESBL activity mostly affect the omega-loop of SHV, including substitutions at Ambler position 179 (SHV-8 [13] and SHV-24 [14]) or 169 (SHV-57 [15]) and an insertion at 163 (SHV-16 [16]). The first variant displaying resistance to BLI (clavulanate and tazobactam) was SHV-10, which owes its unique phenotype to a substitution at codon 130 [17]. Other reported BLI-resistant substitutions are located at Ambler codons 69 (SHV-49 [18]), 234 (SHV-56 [19] and SHV-72 [20]) and 235 (SHV-107 [21]).

In the sequencing era, new *bla*_SHV_ alleles are frequently reported and now number in the hundreds (as of November 2023, up to *bla*_SHV-232_ have been assigned by the NCBI’s Reference Gene Catalog [22], https://www.ncbi.nlm.nih.gov/pathogens/refgene/). Most of these alleles are also catalogued in the β-lactamase database (BLDB) [23] and the Comprehensive Antibiotic Resistance Database (CARD) [11]. Additions to these databases are based on novel aa sequences, without a requirement for biochemical characterization of enzyme function [10,24]. Database curators attempt to assign β-lactamase alleles to the functional groups according to their reported spectrum of activity: narrow spectrum, extended-spectrum (ESBL) and/or BLI resistant. Unfortunately, the primary literature used to support the functional classifications varies widely in terms of experimental design. In some cases, the presence of a *bla*_SHV_ allele in a *K. pneumoniae* isolate displaying ESBL activity has been used to ascribe ESBL functionality to the SHV enzyme, without ruling out the presence of other ESBL enzymes. This has led to the assignment of chromosomal variants with WT activity being reported in the literature as ESBL variants [25] (e.g. SHV-27 and SHV-41), where the error propagated to multiple antimicrobial resistance (AMR) gene databases (in November 2023, SHV-27 and SHV-41 were still recorded as ESBL in BLDB and CARD, despite the error being reported in a 2006 publication [25]). Other examples are summarized in Table S1 (see column ‘Evidence’ for a discussion of discrepancies between databases).

Neubauer *et al.* [26] recently sought to clarify the role of specific substitutions in SHV functionality by systematically reconstructing isogenic mutants carrying individual substitutions (identified in naturally occurring variants with modified activity). Mutations were introduced into the *bla*_SHV-1_ background, and the spectrum of enzyme activity was assessed in an *E. coli* strain lacking any other β-lactamase [26]. This confirmed the role of some substitutions in conferring ESBL activity (at Ambler site 238) or BLI resistance (at Ambler sites 69, 234 and 240); however, some mutants could not be generated. In 2021, we used these findings, together with a review of experimental evidence from the literature, to systematically assign *bla*_SHV_ alleles to functional classes. We also incorporated the resulting *bla*_SHV_ database and list of functionally relevant mutations in the Kleborate tool (v2.0) for genotyping of *K. pneumoniae* genomes [27].

Here, we aimed to systematically assess the evidence for enzyme activity by exploring genotype–phenotype relationships for naturally occurring *bla*_SHV_ alleles using a diverse set of 3999 *K. pneumoniae* isolates with matched genome–phenotype data, which lack non-*bla*_SHV_ alleles. We also explored the evolutionary relationships and genetic context of *bla*_SHV_ alleles, with the aim of further clarifying the emergence and spread of SHV variants.

## Methods

### SHV reference database

**Fig. 1.**
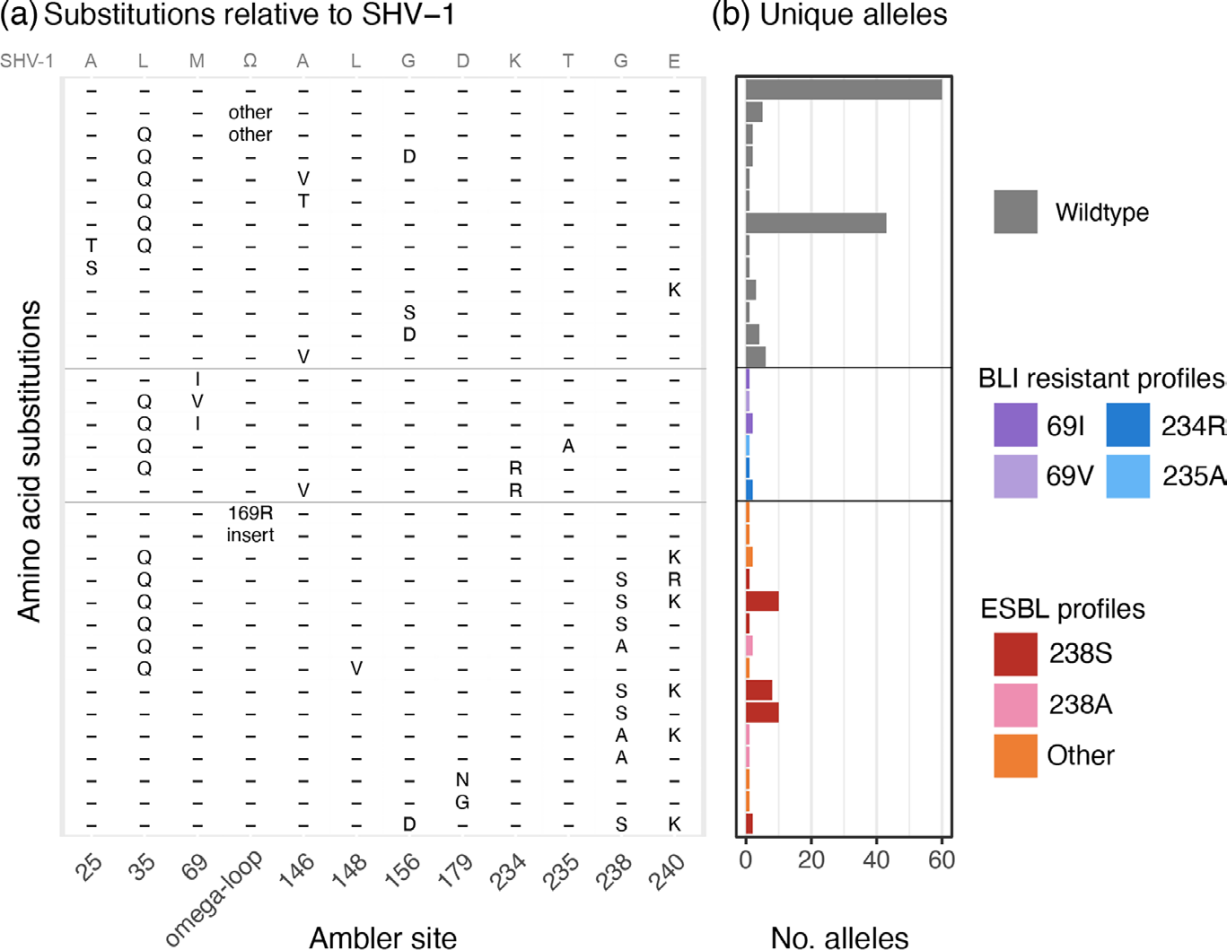
Amino acid substitution profiles associated with *n* = 181 bla_SHV_ alleles. (a) Positions studied in the article by Neubauer *et al.* and tracked in Kleborate v2 are shown as columns; these include sites where substitutions have a clear association with ESBL activity [148, 179, 238 and 240, omega-loop (positions 164–178)] or BLI resistance activity (69, 234 and 235), plus some sites (25, 35, 146 and 156) that are associated with increased MIC to ceftaroline but not ceftriaxone or inhibitor resistance. Position 179 is also a part of the omega-loop and is specifically separated to show its association with ESBL profiles. Position 130 is not included, as it is found only in SHV-10 (BLI resistant) for which there is no nt sequence available. Each row indicates a unique combination of aas across these variable sites. The aas present in SHV-1 are indicated in grey at the top of the panel, where the omega-loop sequence (Ω) is RWETELNEALPGDARD. (b) The number of unique nt alleles associated with each aa profile (row) is shown as a barplot. Colours indicate the functional class assigned to these alleles, on the basis of the mutations shown here and supporting literature (cited in the text and in Table S1).

We curated an updated set of *bla*_SHV_ nt alleles for Kleborate based on a comparison of CARD (v3.2.8) [11], the NCBI’s Reference Gene Catalog [22] and BLDB (as of November 2023, up to allele number *bla*_SHV-228_). *Bla*_SHV-6_ and *bla*_SHV-10_ were excluded as the published nt sequence for *bla*_SHV-6_ is incomplete, and there is no nt sequence for *bla*_SHV-10_ (only an aa sequence for SHV-10, which yields no exact matches to any six-frame translations of nt sequences in the NCBI using tblastn). *Bla*_SHV-11_, *bla*_SHV-28_ and *bla*_SHV-31_ were represented by two nt sequences each (labelled .v1 and .v2) and the rest by a single nt sequence. In addition to reporting matches to known alleles and the corresponding functional class, Kleborate specifically checks for and reports mutations of known functional relevance (listed in Fig. 1; plus Ambler position 130, which was found in two novel alleles and was reported as responsible for BLI resistance in SHV-10 [17]).

We used the rules previously established for Kleborate (v2.0), as described in Supplementary Note 3 of Lam *et al.* [27], to assign functional classes (WT, ESBL or BLI resistant). This relied primarily on the presence of well-supported SHV substitutions as noted above (ESBL: mutation at Ambler sites 238, 179 and 169; BLI resistance: mutation at sites 69, 130 and 234), or direct experimental evidence for individual *bla*_SHV_ alleles (detailed in Results). The curated set of allele sequences and their assignment to functional classes is included in a new release of Kleborate (v2.4.1, DOI: 10.5281/zenodo.10469001) and Table S1. Table S1 also includes information on each allele extracted from the NCBI’s Reference Gene Catalog (including PubMed ID, subclass, protein and nt accessions), BLDB (including phenotype, sequence accessions and alternative names) and CARD (Antibiotic Resistance Ontology identifiers and sequence accessions).

Our curated class assignments were compared with those of BLDB and the NCBI’s Reference Gene Catalog, which were interpreted as follows: WT (phenotype ‘2b’ in BLDB and subclass ‘BETA-LACTAM’ in the NCBI), ESBL (phenotype ‘2be’ in BLDB and subclass ‘CEPHALOSPORIN’ in the NCBI) and BLI resistant (‘2br’ in BLDB and subclass ‘BETA-LACTAM’ in the NCBI but with ‘product name’ typically including the prefix ‘inhibitor resistant’). Discrepancies between our class assignments and those of BLDB or the NCBI’s Reference Gene Catalog are reported in Table S1, which includes a summary of evidence based on the literature review and the phenotype data presented in this study.

Novel alleles, each encoding for unique aa sequences, identified in our sequence data (described below) were submitted to the NCBI’s Reference Gene Catalog to obtain allele numbers in November 2023. These *bla*_SHV_ alleles (encoding SHV-233 to 237 and SHV-239 to 243), together with 12 additional *bla*_SHV_ alleles present in the NCBI in November 2023 but not yet in our database (encoding SHV-115, SHV-116, SHV-132, SHV-146, SHV-171, SHV-190, SHV-191, SHV-202 and SHV-229 to 232), were also added to Kleborate v2.4.1. We also updated the database to include a single representative sequence per allele in Kleborate v2.4.1. For *bla*_SHV-11_, we selected GenBank accession AY293069 (as per BLDB, labelled .v1 in earlier versions of Kleborate), as this sequence is the correct length, and we confirmed exact matches in >1000 of our *K. pneumoniae* genomes in >300 multi-locus sequence types (STs), whilst .v2 was not detected. In the NCBI’s Reference Gene Catalog and CARD, *bla*_SHV-11_ is represented by a different sequence (GenBank accession X98101.1) that has additional nts at the start and end, differs from AY293069 at two synonymous mutations and had no exact matches in *K. pneumoniae* whole genomes. For *bla*_SHV-28_, we used GenBank accession AF299299.1 (as per CARD and BLDB; formerly .v2 in earlier versions of Kleborate), and for *bla*_SHV-31_, we used GenBank accession AY277255.2 (as per the NCBI’s Reference Gene Catalog, CARD and BLDB; formerly .v2 in Kleborate).

### Matched genome and phenotype data for *K. pneumoniae* species complex isolates

A global collection of *K. pneumoniae* species complex genomes with matched antimicrobial susceptibility testing (AST) data was aggregated by the KlebNET-GSP AMR Genotype-Phenotype project group (summarized in Tables S2–S4). This collection includes human, animal and environmental isolates collected between 2001 and 2021 from 24 countries across six continents. Genomes were assembled from Illumina reads using Unicycler (v0.4.8) (accessions and assembly metrics in Table S2) and analysed using Kleborate (v2.2.0) (results in Table S3), which identifies the presence of acquired resistance genes/alleles including SHV, SHV protein variants and porin defects associated with AMR [28] (i.e. loss of OmpK35 or OmpK36 and insertions in loop 3 of OmpK36). All assemblies met the pre-agreed KlebNET-GSP criteria of <5% contamination (assessed using KmerFinder [29] (v3.2)): Kleborate-designated species match of ‘strong’, ≤500 contigs; genome size 4 969 898–6 132 846 bp; and G+C content in the range 56.35–57.98%. Contig metrics across the dataset were as follows: contig count, mean 131.5 and sd 83.1; N50, mean 376 272.4 bp and sd 557 147.6 bp; genome size, mean 5 418 671.9 bp and sd 163 789.2 bp; and G+C content, mean 57.3% and sd 0.2%. We included only *K. pneumoniae* genomes with an exact aa sequence match to one or more known *bla*_SHV_ alleles and excluded those in which other (i.e. non-SHV) β-lactamases were detected (total *n* = 3999 isolates for analysis).

The available AST data were determined by the contributing laboratories using a range of methods, including disc diffusion, agar dilution, broth microdilution and semi-automated methods (Vitek 2 or Phoenix), that were performed based on Clinical and Laboratory Standards Institute (CLSI) or EUCAST guidelines. AST data were shared in the form of disc diffusion zone sizes or minimum inhibitory concentrations (MICs). We interpreted as ‘susceptible’ (S), ‘intermediate/susceptible, increased exposure’ (I) or ‘resistant’ (R) using the EUCAST (v13.0) or CLSI (M100 33rd edition) breakpoints, as appropriate to the assay used. As there were very few isolates categorized as I, we grouped I/R (i.e. non-WT) together and refer to this group as ‘resistant’ (data in Table S4).

### Phylogenetic analysis

*Bla*_SHV_ nt sequences were aligned using MAFFT [30] (v6.861). Pairwise distances between aligned nt sequences were calculated using the ‘dist.dna’ function in the ‘ape’ package (v5.7-1) for R (v4.2.3), and a phylogeny was inferred using the BioNJ algorithm in the same package. The minimum spanning tree was inferred using GrapeTree [31] (v1.5.0) using MSTreeV2. R packages ggplot2 (v3.4.4) and ggtree [32] (v3.6.2) were used for data visualization.

### Genomic context of SHV alleles

The genomic location (chromosome or mobile) of *bla*_SHV_ allelic variants was determined using a combination of literature review; the CARD Prevalence, Resistomes and Variants database (v3.0.9); and blastn (100% nt identity and coverage) searches of *bla*_SHV_ allelic variants in publicly available complete genome sequences and assembly graphs of *K. pneumoniae*. At the time of the search, CARD Prevalence, Resistomes and Variants [11] (v3.0.9, accessed October 2021) included 874 and 5466 *K. pneumoniae* chromosomes and plasmids, respectively, from NCBI genomes. The presence of *bla*_SHV_ alleles amongst these chromosomes and plasmids was extracted from the CARD database. Plasmer (v0.1) [33] was used to predict the genomic locations of blaSHV in the KlebNET-GSP collection. In addition, complete *K. pneumoniae* genomes (*n* = 1296 chromosomes and *n* = 4217 plasmids) were downloaded from the NCBI using ncbi-genome-download (v0.3.1) to directly examine the genomic context of *bla*_SHV_ alleles. We used Kleborate [27] (v2.2.0) to search for SHV variants across complete *K. pneumoniae* genomes from the NCBI and the KlebNET-GSP collection.

To investigate the genetic context of mobile *bla*_SHV_ variants (represented in this analysis by *bla*_SHV-2_, *bla*_SHV-12_ and *bla*_SHV-30_), we used a subset of the publicly available complete genome sequences of *K. pneumoniae* from the NCBI. We selected 30 random genomes from different STs [determined by Kleborate (v.2.2.0)] and ran Mauve [34] (v2015-02-25) to identify the collinear block containing *bla*_SHV_. We then used blastn to search for this collinear block in all publicly available complete chromosome sequences of *K. pneumoniae* from the NCBI, to confirm its broader conservation. To visualize the genetic context of mobile variants of *bla*_SHV_ compared with the typical chromosomal context from which they were presumably mobilized, we extracted 10 kbp of sequence upstream and downstream of the gene from genomes CP103302.1 (*bla*_SHV-2_), NC_009650.1 (*bla*_SHV-12_), NZ_CP017936.1 (*bla*_SHV-30_) and NZ_CP032170.1 (*bla*_SHV-30_). We then used Prokka [35] (v1.14.6) and clinker [36] (v0.0.24) to annotate and visually compare the extracted genomic regions with a representative sequence of the chromosomal collinear block extracted from the chromosome of *K. pneumoniae* strain MGH 78578 (accession CP000647.1). In addition, we used flankophile [37] (v0.2.10) to extract *bla*_SHV_ flanking regions (5 kbp upstream and downstream) across the previously used NCBI’s publicly available complete chromosome *K. pneumoniae* sequences to capture genetic variation in *bla*_SHV_ flanking regions. We then used CD-HIT-EST [38] (v4.8.1) to cluster the flanking regions with ≥90% nt sequence similarity. We used the same visualization methods as described above. We also investigated the presence of insertion elements 10 kbp upstream of WT *bla*_SHV_ in the genomes of phenotypically 3GC resistant isolates using the blastn and the ISfinder [39] database.

For the set of complete genomes, the *bla*_SHV_ copy number was calculated based on the number of unique non-overlapping blastn hits. For the matched genotype–phenotype dataset, the copy number of *bla*_SHV_ in draft genomes was estimated by analysing Illumina read sets, calculating the ratio of read depth for *bla*_SHV_ vs the mean read depth of the seven *K. pneumoniae* loci used for multi-locus sequence typing and using SRST2 [40] (v0.2.0) to perform the mapping and depth calculations. The copy number estimates are included along with other genotype information in Table S3.

## Results

### Distribution of activity-modifying mutations

In Kleborate v2.2.0 database, there are a total of 181 unique *bla*_SHV_ alleles, corresponding to 178 unique protein sequences or variants (Table S1). Figure 1 illustrates the distribution of key aa substitutions (hereafter the term mutations is used for both nt and aa variation for convenience, even though aa changes are a consequence of the actual mutations) across these alleles. Amongst the *bla*_SHV_ alleles identified, 38 encoded mutations relative to SHV-1 at Ambler positions 238 (*n* = 36) and 179 (*n* = 2). These specific mutations have been observed by Neubauer *et al.* [26] to confer 3GC resistance, classifying these variants as ESBL. Five additional protein variants were assigned as ESBL based on primary literature reports (Table S1): SHV-16 [16] (omega-loop insertion between Ambler sites 167 and 168), SHV-57 [15] (omega-loop substitution 169R), SHV-31 (encoded by divergent alleles SHV-31.v1 and SHV-31.v2 [41], each carrying mutations 35Q and 240K) and SHV-70 [42] (mutation 148V). Eight alleles harboured a substitution at a site associated with BLI resistance [26] and were classified accordingly: Ambler site 69 (SHV-49 [18], SHV-52, SHV-92 and SHV-203) or 234 (SHV-56 [19], SHV-72 [20] and SHV-73). SHV-107 (harbouring mutation 235A) was also classified as BLI resistant based on primary literature [21]. The remaining 130 alleles were assigned as WT.

### Evolutionary relationships and genomic context

**Fig. 2.**
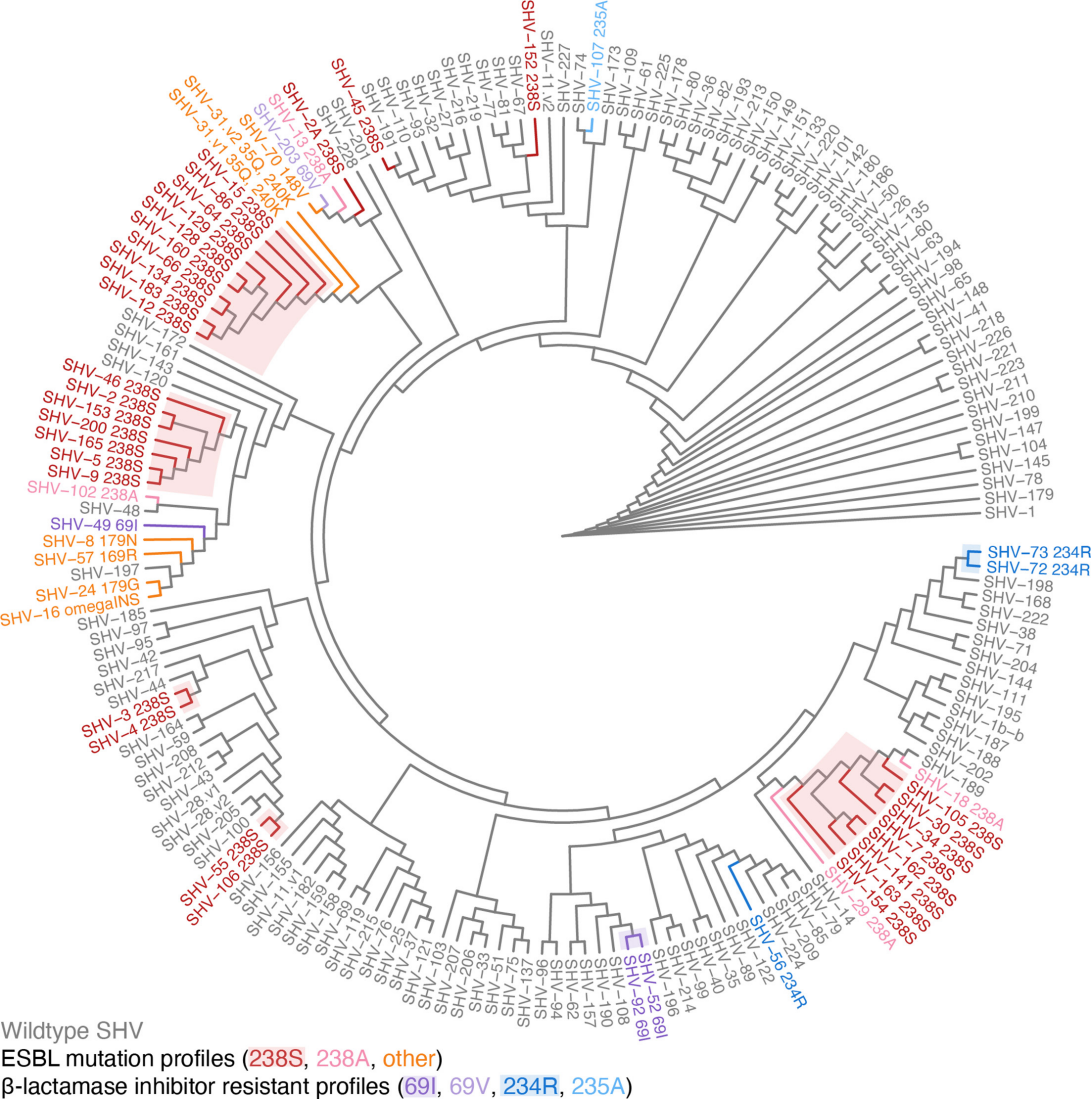
Cladogram for *n* = 181 bla_SHV_ alleles. The cladogram was inferred from a pairwise genetic distance matrix calculated from nt sequences using BioNJ, rooted on SHV-1. Tips are labelled with the SHV allele name and coloured to indicate the mutation profile (black, WT; red, orange and pink, ESBL profiles; and blue and purple, BLI-resistant profiles). For alleles classed as non-WT, the class-modifying mutation is included in the label (e.g. 238S indicates substitution of serine at Ambler site 238 in the encoded protein; ‘omegaINS’ refers to a 6-aa insertion in the omega-loop between Ambler codons 167 and 168). Shading indicates clusters of alleles referred to in the text, which may share class-modifying mutations via vertical inheritance.

**Fig. 3.**
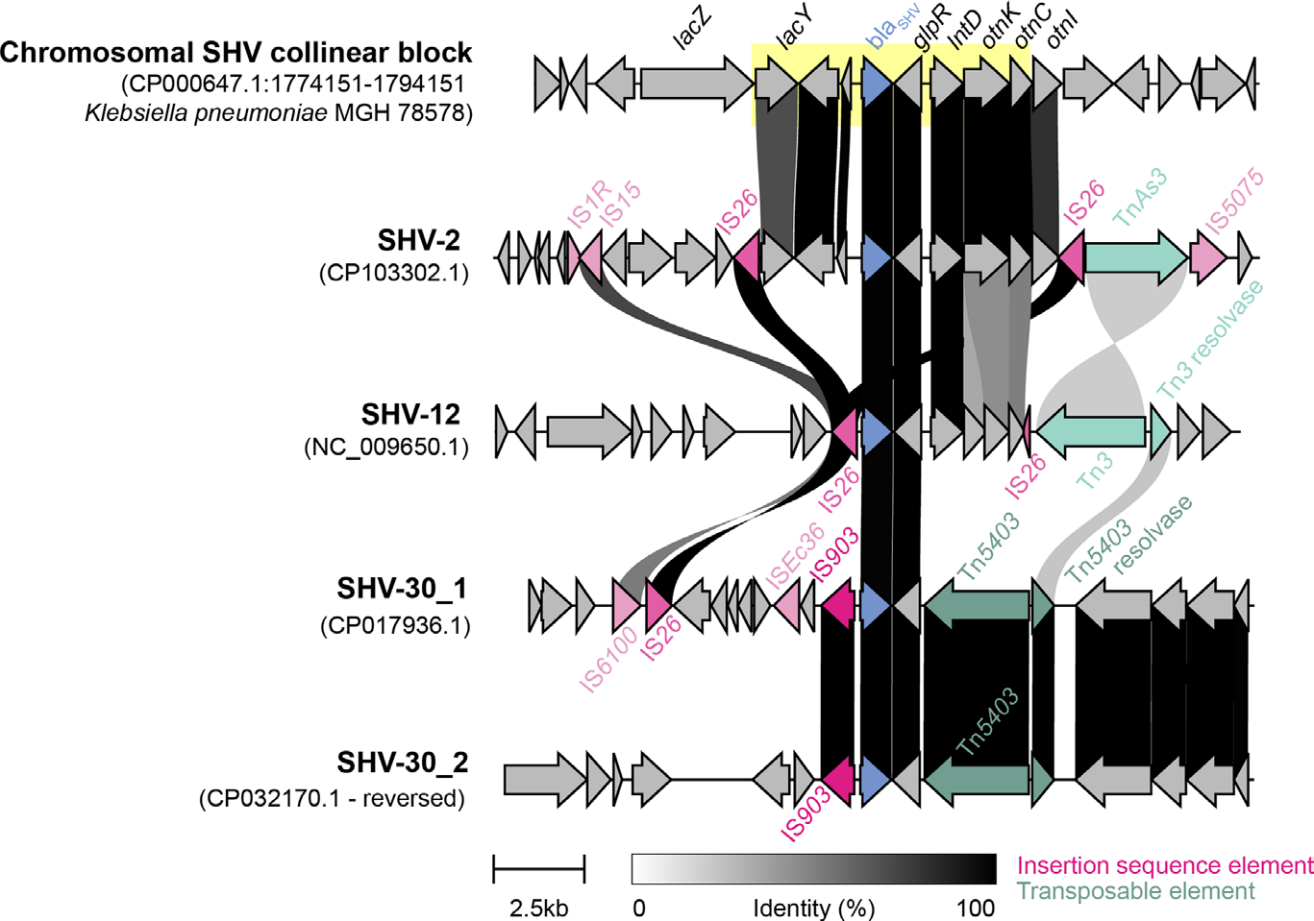
A comparison of the genomic context of SHV allele clusters. Upstream (10 kbp) and downstream (10 kbp) sequences of each bla_SHV_ were extracted and aligned. The 7585 bp chromosomal SHV collinear block is highlighted in yellow. Bla_SHV_ is coloured in blue, while mobile genetic elements, such as insertion sequences and transposons, are illustrated in pink and green, respectively. Percent identity between the genes is shown by the gradient scale bar.

To understand the evolutionary relationships between *bla*_SHV_ alleles, we inferred a cladogram and minimum spanning tree from the nt sequence alignment (Figs 2 and S1). Pairwise genetic distances between allele sequences support *bla*_SHV-1_ as the ancestral form, as it has the smallest distance to all other variants (mean 5.1 substitutions and total distance 918; compared with mean 5.4 and total 969 for *bla*_SHV-11_, which had the next lowest values). We therefore rooted the phylogeny at *bla*_SHV-1_. We identified a chromosomal *bla*_SHV_ collinear block (7585 bp) conserved in 90.5% (*n* = 1236/1366) of complete *K. pneumoniae* genomes with >90% nt identity and >90% coverage (Fig. 3). In general, there is a low genetic variation of the chromosomal *bla*_SHV_ flanking regions (5 kbp upstream and downstream), where 95.1% (*n* = 1229/1292) of complete genomes had ≥90% nt sequence similarity in their flanking regions. Comparing the chromosomal *bla*_SHV_ collinear block with the genomic context of plasmid-borne and IS*26*-mediated *bla*_SHV-2_ and *bla*_SHV-12_, the chromosomal *bla*_SHV_ collinear block is conserved in the genomic context of *bla*_SHV-2_ and flanked by IS*26*. Similarly, the chromosomal *bla*_SHV_ collinear block is partially conserved (59% coverage and 99% identity of 7585 bp) in the *bla*_SHV-12_ genomic context. The gene directly downstream of *bla*_SHV_, *glpR*, encodes a glycerol-3-phosphate regulon repressor, which is conserved across the genomic contexts of chromosomal and plasmid-borne *bla*_SHVs_.

ESBL alleles were distributed throughout the cladogram (pink, red and orange in Fig. 2), consistent with at least 19 independent mutation events [*n* = 12 in Ambler codon 238, *n* = 1 in codon 148, *n* = 2 in codon 179, *n* = 2 others in the omega-loop and *n* = 2 (SHV-31.v1 and SHV-31.v2) in codon 240] [41]. Some ESBL alleles formed clusters that appear to share a resistance-conferring mutation (238S) via inheritance from a common ancestor (shading, Fig. 2). These include two pairs of alleles (*bla*_SHV-3_/*bla*_SHV-4_ and *bla*_SHV-55_/*bla*_SHV-106_) and three larger clusters centred around *bla*_SHV-2_/*bla*_SHV-5_ (*n* = 7), *bla*_SHV-12_ (*n* = 10) and *bla*_SHV-7_/*bla*_SHV-30_ (*n* = 8). Mobilization of *bla*_SHV-2_ and *bla*_SHV-12_ by IS*26* are well documented [1,10]. *Bla*_SHV-3_ and *bla*_SHV-4_ are also known to be plasmid borne [43] and found in species outside *Klebsiella* [10]*,* although we could not identify a complete plasmid sequence in which to explore the specific genetic context of the mobilized region. The members of the *bla*_SHV-7_ cluster, including *bla*_SHV-7_ [44], *bla*_SHV-30_ [45] and *bla*_SHV-34_ [46], have been reported as plasmid borne and found in *Enterobacter*. In the KlebNET-GSP collection, *bla*_*SHV-7*_ (*n* = 8) was identified in seven plasmids and one chromosome, whilst *bla*_*SHV-154*_ (*n* = 1) was identified in a chromosome (Table S5). Amongst the *bla*_SHV-7_ cluster, only *bla*_SHV-30_ was detected amongst complete *K. pneumoniae* genome sequences in the NCBI. This allele was identified in two similar plasmid sequences [99.99% identity over 55 821 bp of shared sequence (85% coverage), detected in ST2938 (accession NZ_CP032170.1) and ST45 (accession NZ_CP017936.1)], where it was flanked by IS*903* and Tn*5403* (Fig. 3). This provides a potentially novel, non-IS*26*-mediated, mobility mechanism for this ESBL cluster, which was found in a total of 13 *K. pneumoniae* genomes belonging to seven STs (and one *K. variicola* genome) in our genome collection. We could find no evidence to support that *bla*_SHV-55_/*bla*_SHV-106_ have been mobilized out of the *K. pneumoniae* chromosome. NCBI blast did not identify these alleles outside *K. pneumoniae*, nor in any complete *K. pneumoniae* genomes. We identified a single instance in our genome collection (*bla*_SHV-106_ in an ST14 genome); read analysis indicated a copy number of one, suggesting this was the only copy of *bla*_SHV_ in the genome, and assembly graph analysis and plasmid prediction analysis supported its location in the chromosome (Table S5). Four of the five ESBL allele clusters therefore appear to be plasmid borne and likely reflect the diversification of ESBL alleles following mobilization from the *K. pneumoniae* chromosome.

BLI-resistant alleles (*n* = 8) were distributed throughout the cladogram (blue and purple in Fig. 2), consistent with at least six independent mutation events (*n* = 3 in Ambler codon 69, *n* = 2 in 234 and *n* = 1 in 235). These alleles have not been reported outside of *K. pneumoniae*, and sequence searches of the NCBI and CARD did not detect evidence of them in non-*K. pneumoniae* genomes. The original reports of *bla*_SHV-56_ and *bla*_SHV-49_ confirmed these variants as chromosomally located [19], and we also found *bla*_SHV-52_ and *bla*_SHV-56_ in draft genome sequences where assembly graph inspection and plasmid prediction software confirmed that they were located on chromosomal contigs (Table S5). The other alleles *bla*_SHV-72_, *bla*_SHV-73_ and *bla*_SHV-92_ were not found in our genome collection or in NCBI genomes via blastn search. The original report of *bla*_SHV-92_ states that it was detected in a transconjugant, suggesting that it was plasmid borne [47]; however, we found no evidence of any other BLI-resistant alleles being mobile. These data suggest that the currently reported BLI-resistant *bla*_SHV_ alleles have arisen in WT chromosomal *bla*_SHV_ backgrounds. With the exception of *bla*_SHV-92_, these BLI-resistant alleles have not yet been mobilized to plasmids, which is consistent with the low prevalence of the phenotype reported in *K. pneumoniae* isolates.

### Genotype–phenotype relationships

We compared *bla*_SHV_ alleles with AST phenotypes for 3GCs and BLIs in a set of *n* = 3999 *K. pneumoniae* genomes that carried at least one *bla*_SHV_ allele and no other β-lactamase (Tables S2–S4). Within these genomes, we identified 70 of the known 181 *bla*_SHV_ alleles [38% of those in the Kleborate (v2.2.0)].

**Fig. 4.**
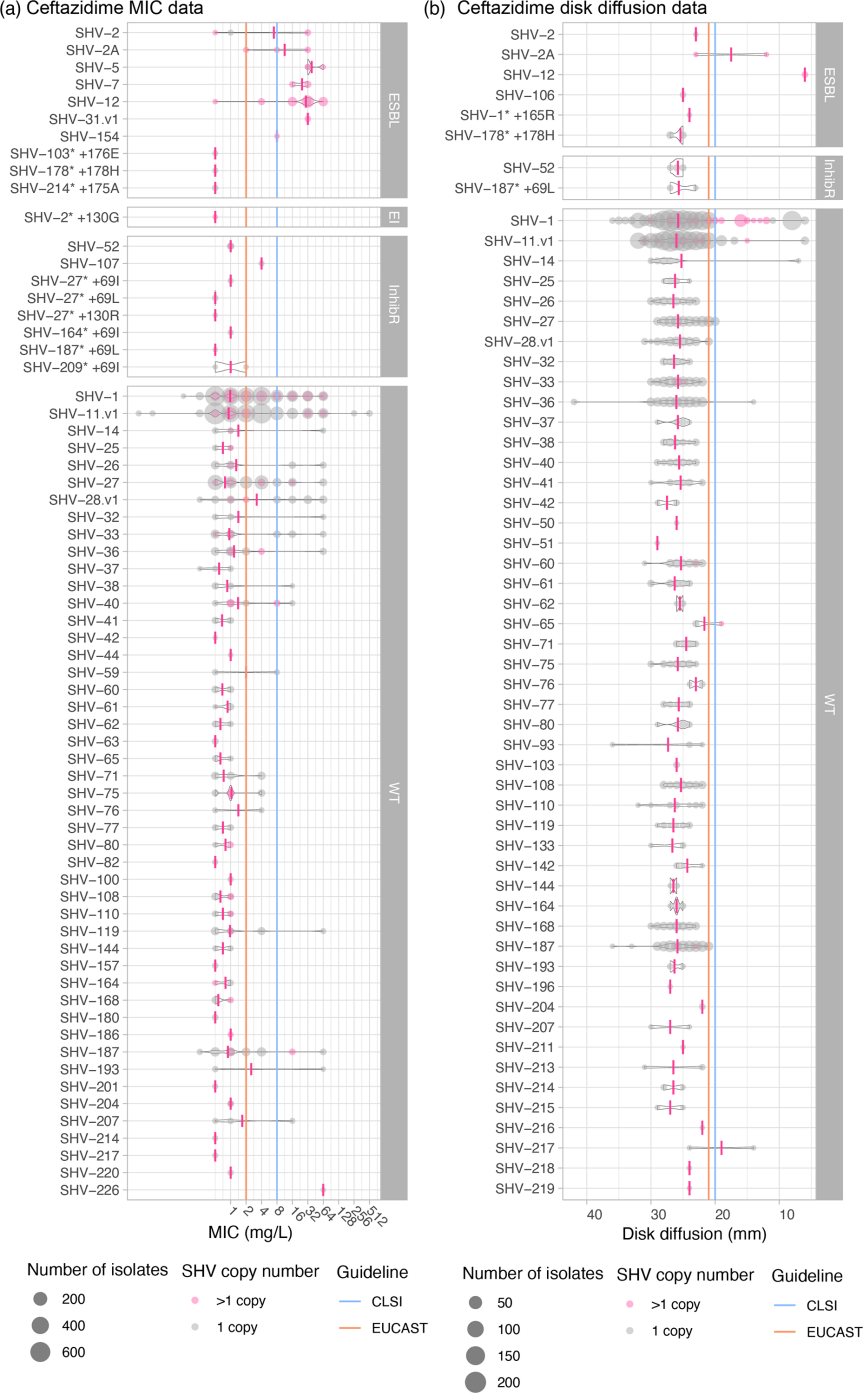
AST value distributions for ceftazidime. The size of each circle represents the number of isolates with an SHV allele and no other acquired β-lactamase. (a) MIC and (b) disc diffusion measurements show the distribution of phenotypes for each SHV allele. Average MIC and disc diffusion measurements per SHV allele are indicated by a pink vertical line. Grey circles indicate 1 SHV copy, whilst pink circles indicate >1 SHV copy. SHV alleles are grouped based on ESBL, ESBL- and BLI resistant (EI), BLI resistant (inhibR) and WT phenotype classifications. EUCAST (v13.0) or CLSI (M100 33rd edition) S/I breakpoints are indicated using orange and blue lines, respectively. SHV-187* +69L is SHV-132 in Kleborate v2.4.1. For MIC values, larger values indicate increased resistance; for disc diffusion results, larger zone sizes indicate increased susceptibility.

Eight known ESBL protein variants (classified as such in the literature and here) were identified in isolates that were tested for susceptibility to ceftazidime and all but one (sole representative of SHV-106) showed evidence for resistance (see Table 1, Fig. 4). All of these protein variants have at least a 238S substitution, with the exception of SHV-31.v1, which had both 35Q and 240K substitutions. All isolates representing the remaining protein variants and for which data were available also showed evidence of resistance to ceftriaxone. However, resistance to cefotaxime was more variable. Eleven other isolates carried *bla*_SHV_ alleles with a non-synonymous mutation in the omega-loop but were not previously reported as ESBL alleles (*bla*_SHV-51_ and four novel alleles, see Table 1); all tested susceptible to ceftazidime (Table 1, Fig. 4) and all other 3GCs for which they were tested (Table 1).

**Table 1.** 3GC susceptibility phenotypes for ESBL-assigned alleles

Allele	Ceftazidime (*R*/*n*, %*R*)	Cefotaxime (*R*/*n*, %*R*)	Ceftriaxone (*R*/*n*, %*R*	Mutation/s
Exact matches to known alleles assigned here as ESBL				
SHV-2	3/6 (50%)	1/2 (50%)	3/3 (100%)	238S
SHV-2A	5/6 (83.3%)	2/3 (66.7%)	2/2 (100%)	35Q, 238S
SHV-5	4/4 (100%)	1/1 (100%)	3/3 (100%)	238S, 240K
SHV-7	8/8 (100%)	–	8/8 (100%)	238S, 240K
SHV-12	81/83 (97.6%)	2/2 (100%)	82/82 (100%)	35Q, 238S, 240K
SHV-31.v1	2/2 (100%)	–	2/2 (100%)	35Q, 240K
SHV-106	0/1 (0%)	0/1 (0%)	–	238S
SHV-154	1/1 (100%)	–	1/1 (100%)	238S, 240K
Other alleles with omega-loop mutations				
SHV-1* +165R	0/1 (0%)	0/1 (0%)	–	165R
SHV-103* +176E	0/1 (0%)	–	0/1 (0%)	176E
SHV-178* +178 h	0/6 (0%)	0/6 (0%)	0/1 (0%)	35Q, 178 h
SHV-214* +175A	0/2 (0%)	–	0/2 (0%)	175A
SHV-51 (175A)	0/1 (0%)	0/1 (0%)	–	175A
Exact matches to alleles reported in the literature as ESBL but assigned here as WT				
SHV-27	14/304 (4.6%)	4/106 (3.8%)	12/211 (5.7%)	156D
SHV-38	1/21 (4.8%)	0/14 (0%)	1/7 (14.3%)	146V
SHV-40	3/31 (9.7%)	0/15 (0%)	3/30 (10%)	35Q
SHV-41	0/17 (0%)	0/9 (0%)	1/8 (12.5%)	–
SHV-42	0/3 (0%)	0/3 (0%)	–	25S
SHV-65	1/6 (16.7%)	1/3 (33.3%)	0/4 (0%)	–
SHV-164	0/7 (0%)	0/5 (0%)	0/2 (0%)	–
SHV-187	3/135 (2.2%)	0/87 (0%)	4/51 (7.8%)	–

Novel alleles identified in this study have been highlighted with an ‘*’.

We identified *n* = 533 isolates with alleles that were initially reported as ESBL and assigned as such in the NCBI’s Reference Gene Catalog and/or BLDB but do not carry any causative mutations and were therefore classified in our database as WT (encodes SHV-27, SHV-38, SHV-40, SHV-41, SHV-42, SHV-65, SHV-164 and SHV-187). Our phenotype data support the assignment to WT for all these alleles (see Table 1). A summary of the comparison with BLDB and the NCBI’s Reference Gene Catalog’s class assignments is given in Table S1, available in the online version of this article.

**Fig. 5.**
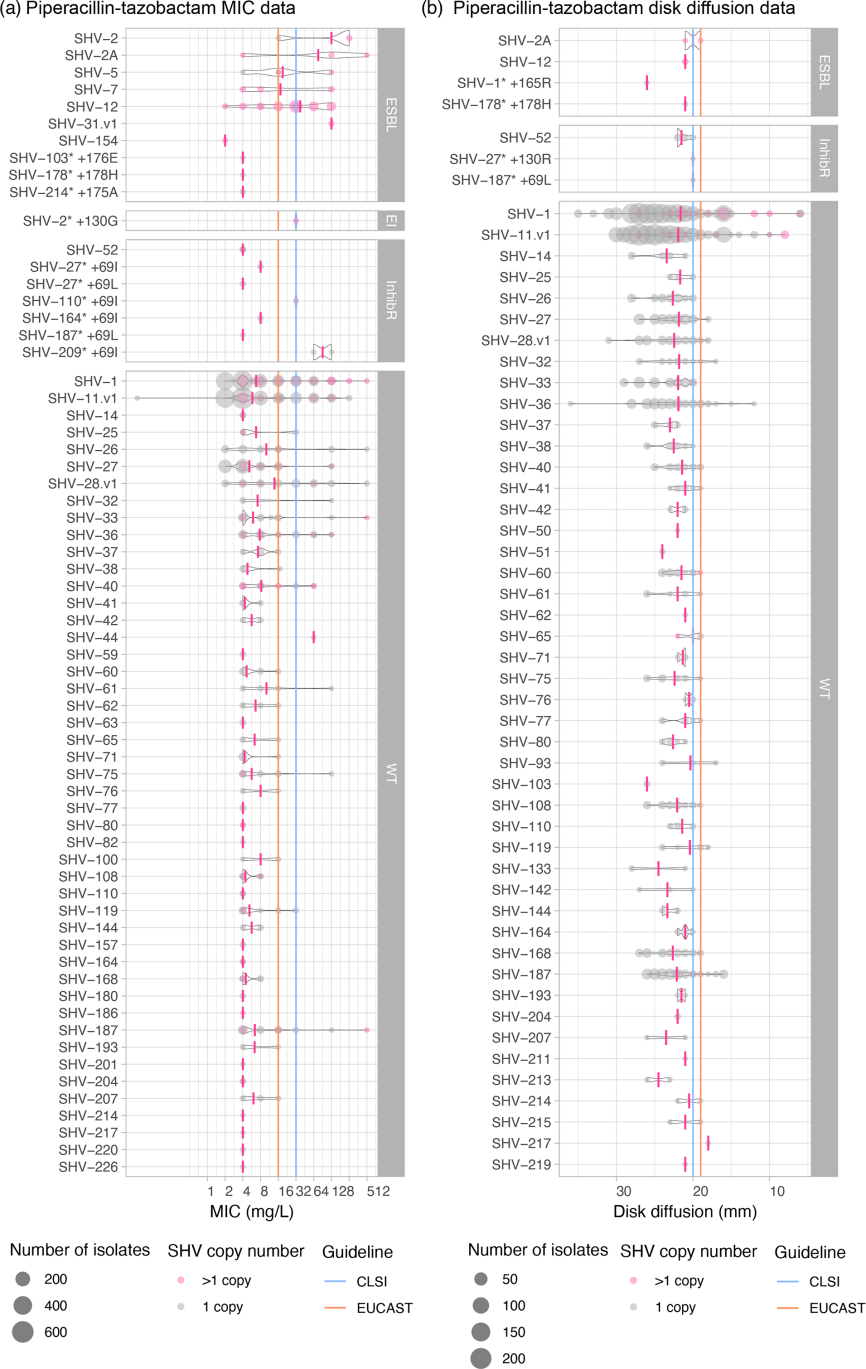
AST value distributions for piperacillin-tazobactam. The size of each circle represents the number of isolates with an SHV allele and no other acquired β-lactamase. (a) MIC and (b) disc diffusion measurements show the distribution of phenotypes for each SHV allele. The average MIC and disc diffusion measurements per SHV allele are indicated by a pink vertical line. Grey circles indicate 1 SHV copy, whilst pink circles indicate >1 SHV copy. SHV alleles are grouped based on ESBL, ESBL and BLI resistant (EI), BLI resistant (inhibR),and WT phenotype classifications. EUCAST (v13.0) or CLSI (M100 33rd edition) S/I breakpoints are indicated using orange and blue lines, respectively. SHV-187* +69L is SHV-132 in Kleborate v2.4.1. For MIC values, larger values indicate increased resistance; for disc diffusion results, larger zone sizes indicate increased susceptibility.

Two BLI-resistant variants were identified in isolates that were tested for susceptibility to piperacillin-tazobactam and/or amoxicillin-clavulanic acid: SHV-52 (which harbours 69I) and SHV-107 (which harbours 235A) (see Table 2). The two isolates carrying SHV-107 came from the same study and were resistant to amoxicillin-clavulanic acid as expected (MIC 32 mg l^−1^ via the automated Vitek platform; piperacillin-tazobactam results were not available). All isolates carrying SHV-52 and tested for piperacillin-tazobactam were susceptible (*n* = 12, from five different studies using either disc diffusion or MIC via Vitek); *n* = 9 of these isolates were also tested for amoxicillin-clavulanic acid, and all were susceptible. The closely related allele SHV-92, which shares the 69I mutation and clusters with *bla*_SHV-52_ in the cladogram (differing from it at a single nt, see Fig. 1), was not present in our dataset, so we could not assess its phenotype directly; the original report of this allele also did not assess phenotype [47]. Nine isolates carried novel variants harbouring a substitution at Ambler site 69; six of these tested susceptible to BLIs and three tested resistant to piperacillin-tazobactam and amoxicillin-clavulanic acid (Table 2, Fig. 5). The resistant isolates were as follows: *n* = 1 carrying a novel variant closest to SHV-110 with additional mutation 69I (accession: SRR15097887) and *n* = 2 (from different studies [48,49], accessions: SRR15098057 and ERR486441) harbouring a novel variant closest to SHV-209 with additional mutation 69I (Tables 2 and S4). Two isolates were identified with novel alleles carrying mutations at codon 130, one (carrying SHV-27 plus 130R) was tested for susceptibility to BLIs but showed susceptibility to both piperacillin-tazobactam and amoxicillin-clavulanic acid via disc diffusion (Table 2, Fig. 5). The other isolate (carrying SHV-2 plus 130G) was resistant to both piperacillin-tazobactam and amoxicillin-clavulanic acid via agar dilution (Table 2, Fig. 5).

**Table 2.** BLI susceptibility phenotypes for inhibitor resistance-assigned alleles

Allele	Piperacillin-tazobactam (*R*/*n*, %*R*)	Amoxicillin-clavulanic acid (*R*/*n*, %*R*)	Mutation/s
Exact matches to known alleles assigned as inhibitor resistant			
SHV-52	0/12 (100%)	0/9 (100%)	35Q, 69I
SHV-107	–	2/2 (100%)	235A
Other alleles with mutations at Ambler site 69, 130, 234 or 235			
SHV-2 +130G	1/1 (100%)	1/1 (100%)	130G, 238S
SHV-27* +69I	0/1 (0%)	0/1 (0%)	69I
SHV-27* +69L	0/2 (0%)	0/2 (0%)	69L
SHV-27* +130R	0/1 (0%)	0/1 (0%)	130R
SHV-110* +69I	1/1 (100%)	1/1 (100%)	35Q, 69I, 156D
SHV-164* +69I	0/1 (0%)	0/1 (0%)	69I
SHV-187* +69L†	0/2 (0%)	0/1 (0%)	69L
SHV-209* +69I	2/2 (100%)	1/1 (100%)	35Q, 69I
Exact matches to alleles reported in the literature as inhibitor resistant but assigned here as WT			
SHV-26	13/63 (20.6%)	10/59 (16.9%)	–

Novel alleles identified in this study have been highlighted with an ‘*’.

†SHV-187* +69L is SHV-132 in Kleborate v2.4.1.

We also identified *n* = 63 isolates carrying SHV-26, which we assigned as WT due to lacking functional mutations but is classified as BLI resistant (2br) in BLDB. The original report of SHV-26 [50] described it as harbouring a mutation at Ambler site 187 and reduced susceptibility to amoxicillin-clavulanic acid (to ‘intermediate/susceptible, increased exposure’ levels). However, this mutation (A187T) was tested by Neubauer *et al.* [26] who found no effect on BLI susceptibility and concluded that the phenotype was likely incorrectly assigned. Our data show some evidence of a BLI-resistant phenotype [(*n* = 13/63 (20.6%) to piperacillin-tazobactam and *n* = 10/59 (17%) resistant to amoxicillin-clavulanic acid], but with majority support for WT (Table 2).

**Fig. 6.**
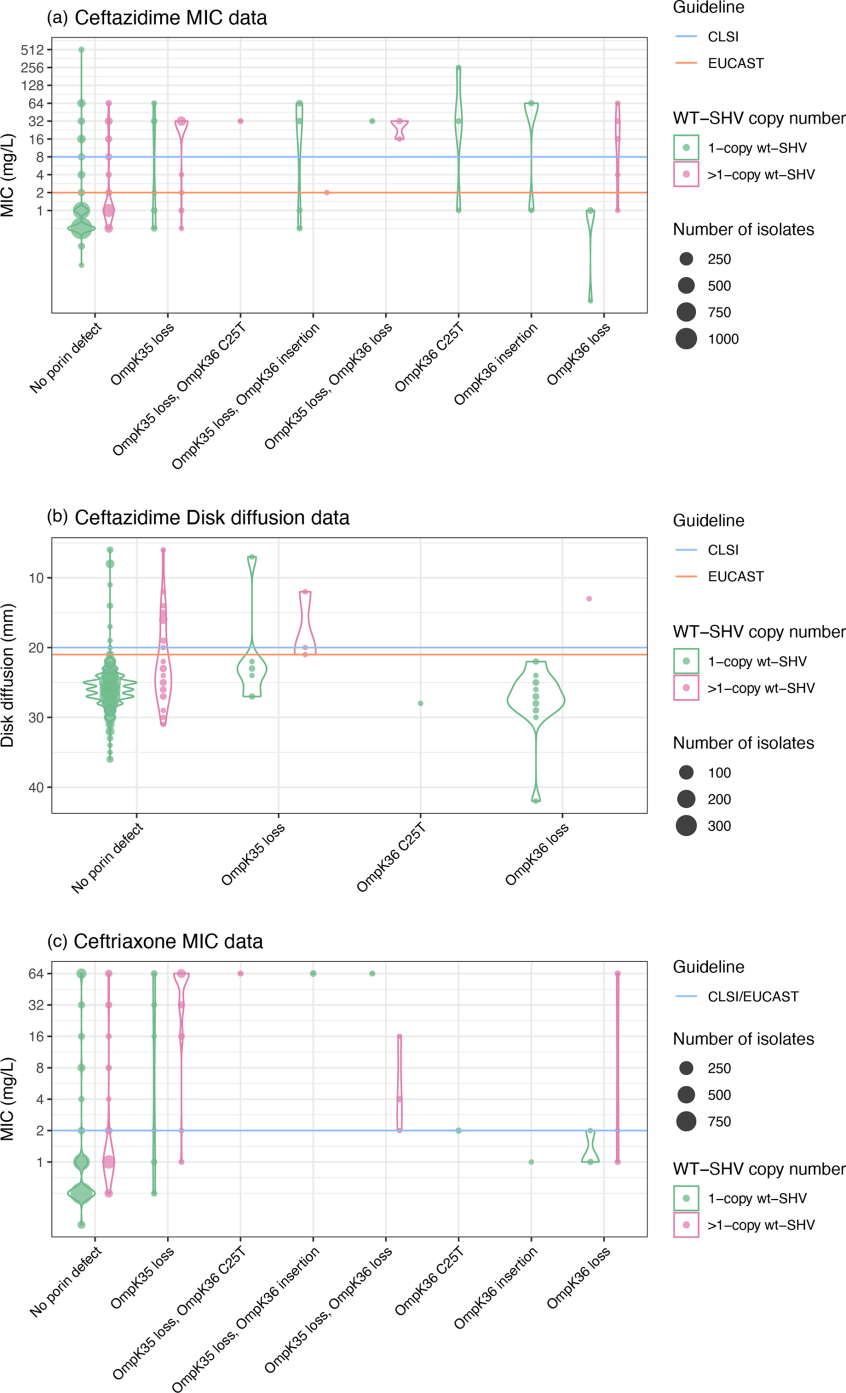
Presence of porin defects and copy number effects amongst isolates with WT-assigned alleles with genomes tested against 3GCs. Violin plots show the distribution of susceptibility testing measures, coloured by copy number, for WT SHV alleles (*n* = 1659 isolates tested against ceftazidime and *n* = 1937 isolates tested against ceftriaxone). EUCAST (v13.0) or CLSI (M100 33rd edition) I/R breakpoints are indicated using orange and blue lines, respectively.

**Fig. 7.**
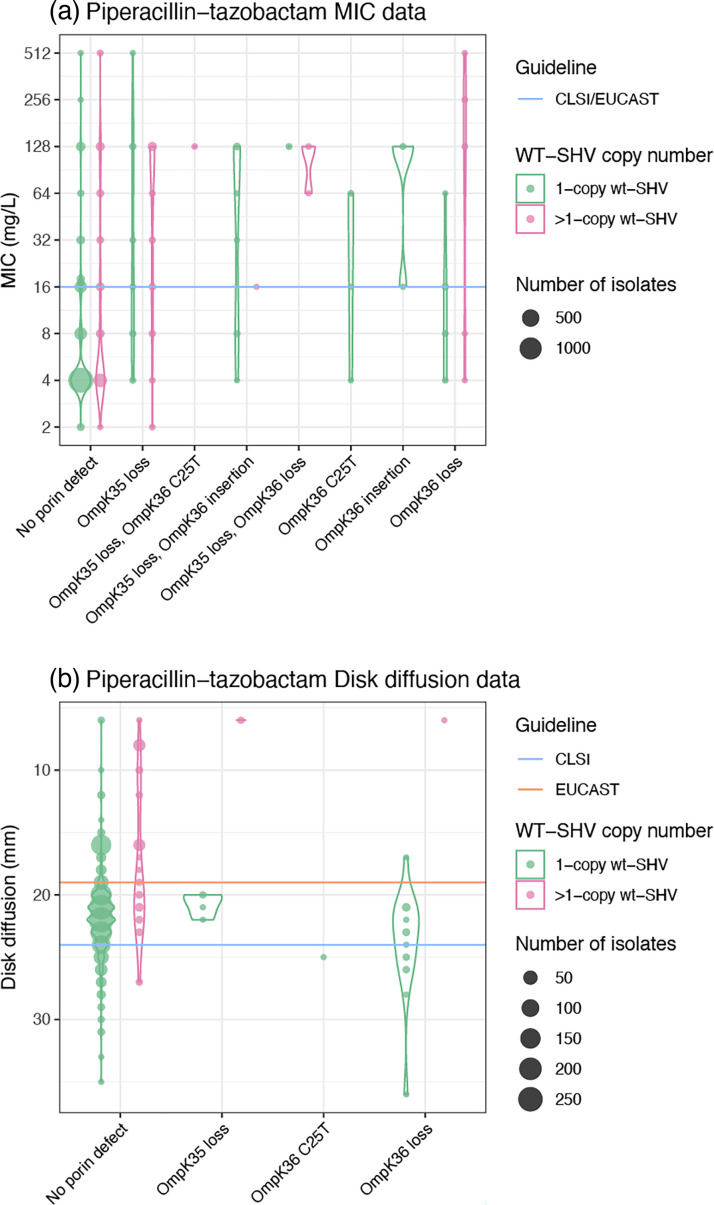
Presence of porin defects and copy number effects amongst isolates with WT-assigned alleles with genomes tested against piperacillin-tazobactam. Violin plots show the distribution of susceptibility testing measures, coloured by copy number, for *n* = 2268 isolates with WT SHV alleles. EUCAST (v13.0) or CLSI (M100 33rd edition) I/R breakpoints are indicated using orange and blue lines, respectively.

Sixty alleles classified as WT were detected in the genome collection (total *n* = 3858 isolates), and the WT phenotype was supported in all cases. Thirty-six of these alleles (60%) were found only in 3GC-susceptible isolates. One allele (*bla*_SHV-59_) was found in one resistant isolate and one susceptible (both ST76 with no other resistance determinants detected). The remaining *n* = 23 WT-classified alleles were primarily found in susceptible strains (66.7–98.0% susceptible, per allele). These include alleles *bla*_SHV-1_ and *bla*_SHV-11_, the most common and well-known WT alleles. We hypothesized that the increased copy number of *bla*_SHV_ and/or porin mutations could explain 3GC and BLI resistance in isolates with WT-assigned *bla*_SHV_ alleles and no other acquired β-lactamases. Amongst isolates with a WT-assigned *bla*_SHV_ allele, *bla*_SHV_ copy number was indeed significantly associated with ceftazidime MIC (correlation = 0.21, *P* < 1×10^−15^ using linear regression on log_2_ MIC), disc diffusion zone diameter (correlation = −0.76, *P* < 1×10^−15^ using linear regression) and clinical resistance (mean 2.7 vs 1.1 copies, *P* = 2×10^−6^ using the Wilcoxon rank sum test). Similarly, *bla*_SHV_ copy number in isolates with WT *bla*_SHVs_ was significantly associated with piperacillin-tazobactam MIC (correlation = 0.25, *P* < 1×10^−15^ using linear regression on log_2_ MIC), disc diffusion zone diameter (correlation = −0.10, *P* < 1×10^−15^ using linear regression) and clinical resistance (mean 2.3 vs 1.1 copies, *P* = 2×10^−6^ using the Wilcoxon rank sum test). The presence of two or more copies of *bla*_SHVs_ was significantly associated with ceftazidime resistance (odds ratio (OR) 3.6, *P* = 6×10^−13^ amongst isolates with a WT-assigned *bla*_SHV_ allele), accounting for 25.6% of the resistance observed amongst these isolates. A further 12.8% of ceftazidime resistance could potentially be explained by porin defects in isolates with a single *bla*_SHV_ copy (see Fig. 6). We also investigated the presence of insertion sequences upstream of WT *bla*_SHV_ (with no other acquired β-lactamases) in genomes of phenotypically 3GC-resistant isolates that could potentially explain the phenotype [3], but there were none identified. For piperacillin-tazobactam, the presence of two or more copies of *bla*_SHV_ was significantly associated with resistance (OR 4.78, *P* < 1×10^−15^ amongst isolates with a WT-assigned *bla*_SHV_ allele), accounting for 34.1% of unexplained resistance, with a further 9.5% potentially explained by porin defects (see Fig. 7).

## Discussion

*Bla*_SHV_ alleles have been studied since their discovery in 1972 and were first explored phylogenetically in 1990 to study the context of *bla*_SHV-2_ and its relationships with other β-lactamase genes [51]. As new *bla*_SHV_ variants are discovered, phylogenetic trees were inferred to explore their ancestry and relationships with each other [2,10, 52]. Most recently, Liakopoulos *et al.* inferred a maximum likelihood tree with 149 SHV-type β-lactamases, but it was unclear which *bla*_SHV_ was the likely ancestral variant [10]. It has been assumed that SHV-1 is the ancestral variant since it was the first *bla*_SHV_ discovered, and our cladogram, pairwise distance data and minimum-spanning tree also support *bla*_SHV-1_ as the ancestral variant (Figs 2 and S1). There is also support from Chaves *et al.* [53] and Haeggman *et al.* [54] who show that *bla*_SHV-1_ is predominantly species specific to * K. pneumoniae* and has a long evolutionary history as a stable chromosomal gene, suggesting that even the ancestor of *bla*_SHV-1_ is also from the *K. pneumoniae* chromosome.

Our phylogenetic and comparative genomic analyses support that ESBL- and BLI-resistant variants of *bla*_SHV_ have evolved multiple times independently through parallel substitution mutations (Fig. 2) and that many of these variants have been mobilized out of the *K. pneumoniae* chromosome via independent events (Fig. 3), enabling them to spread between lineages, species and genera. We found evidence of mobilization for most ESBL variants but only one BLI-resistant conferring variant (SHV-92). Consistent with this, most 3GC-resistant *K. pneumoniae* carrying ESBL variants and no other β-lactamases were found to have multiple copies of SHV (presumably a chromosomal copy with WT activity plus a plasmid-borne copy with ESBL activity).

We have reviewed the classification of *bla*_SHV_ alleles into functional classes to better support the interpretation of genomic data. Our work builds on the experimental study of Neubauer *et al.*, which provided evidence of the role of specific mutations in enzyme activity. By systematically assigning alleles to functional classes based on the presence of specific mutations associated with enzyme activity (Fig. 1), rather than the presence in an ESBL- or BLI-resistant isolate (which may confuse mobile and chromosomal variants), we propose re-classification of 20 *bla*_SHV_ alleles from ESBL to WT (*n* = 12 changes vs the NCBI’s Reference Gene Catalog and *n* = 14 changes vs BLDB, see Table S1).

We used matched genotype–phenotype data, for 3999 *K. pneumoniae* carrying *bla*_SHV_ and no other acquired β-lactamases, to assess the predictability of phenotype based on *bla*_SHV_ alleles (Figs4  5, Tables1  2). For this, we used our Kleborate tool to identify and type *bla*_SHV_ alleles and specific SHV mutations associated with a change in enzyme activity. This analysis provided additional support for the role of 238S and 179G [13,14] in ESBL activity and consequent 3GC resistance but suggests that most changes in the omega-loop do not result in a change in activity. These data also support our classification of variants SHV-27, SHV-38, SHV-40, SHV-41, SHV-42, SHV-65, SHV-164 and SHV-187 – which lack mutations at site 238 or any other mutations associated experimentally with resistance – as WT.

Mutations 69I and 69V have been thought to explain BLI resistance of variants SHV-49, SHV-52, SHV-92 and SHV-203, respectively, and were found by Neubauer *et al.* to confer resistance to piperacillin-tazobactam. Interestingly, our data do not support a simple association between Ambler site 69 mutations and BLI resistance in *K. pneumoniae*, whether in the SHV-52 variant (*n* = 0/12 resistant to piperacillin-tazobactam or amoxicillin-clavulanic acid) or arising *de novo* in other SHV backgrounds (SHV-27, SHV-110, SHV-164, SHV-187 and SHV-209) (*n* = 3/9 resistant). We identified two genomes with a mutation at Ambler site 235 (both SHV-107, which carry mutation 235A) which were both resistant to amoxicillin-clavulanic acid (piperacillin-tazobactam was not tested), providing support for the role of this mutation, which was confirmed by Neubauer *et al.* [26].

The approach and results outlined here have been implemented in Kleborate v2.4.1, along with all new alleles identified in this study, and all those available in public databases as of 7 November 2023. In the Kleborate v2.4.1 database, known *bla*_SHV_ alleles classified as ESBL are those with aa substitutions at Ambler site 238 (*n* = 36 alleles), 179 (SHV-8), 169 (SHV-57), 148 (SHV-70) and 240K+35Q (SHV-31) or insertion in the omega-loop (SHV-16). SHV variants are classified as BLI resistant if they possess mutations at Ambler site 69, 130, 234 or 235. Where exact nt or protein matches are found to a known allele, these are reported in the relevant column (Bla_ESBL, Bla_inhibR, Bla_ESBL_inhibR and Bla_chr) based on the classification in the Kleborate database. As the mutations noted above are considered causative of a change of enzyme activity (class modifying), Kleborate checks for these mutations in all SHV sequences and reports them in a separate column, SHV_mutations. If a class-modifying mutation is detected in an otherwise WT-classified allele background, the novel allele will be reported in the relevant functional column, i.e. Bla_ESBL, Bla_ESBL_inhibR or Bla_inhibR rather than Bla_chr, and labelled with the mutation. Kleborate will also report any mutation in the omega-loop (sites 164–179) in the SHV_mutations column, as it is theoretically possible that any modification disrupting the omega-loop structure could impact function [16,55, 56]. However, the detection of these mutations will not change the class assignment in Kleborate since most changes are likely to be non-functional and all novel omega-loop mutants we identified in our study tested susceptible to 3GCs (Table 2). Our phenotype data also do not support a simple association between mutations at Ambler site 69 and clinical resistance to piperacillin-tazobactam or amoxicillin-clavulanic acid (Table 2). However, our numbers are small (*n* = 9 isolates, of which three tested resistant), and the functional evidence for BLI resistance associated with mutations at this site is convincing [18,26, 57]; therefore, we consider it appropriate to distinguish alleles with Ambler site 69 mutations from WT alleles in the Kleborate database and reporting.

The KlebNET-GSP matched genotype–phenotype dataset yielded coverage of 40% of known *bla*_SHV_ alleles in otherwise β-lactamase-free backgrounds, which is essential to interpret the role of *bla*_SHV_ specifically. Despite other alleles being in unfavourable genomic contexts, our approach enabled a systematic assessment of how *bla*_SHV_ alleles are assigned to functional classes in the public AMR gene databases and provides evidence that some existing assignments are incorrect (Table S1). In turn, this helped us to implement a more transparent and consistent approach to detecting and reporting known and novel *bla*_SHV_ alleles in *K. pneumoniae* genomes, via Kleborate v2.4.1. In addition, the diversity of this dataset (isolates from 24 different countries across 2001–2021 and collected from humans, animals and environments) avoids the very often local epidemiological effects that could bias results. Additional insights into the role of genetic background, expression and co-expression of SHV variants and/or other β-lactamases on resistance mechanisms will help to further clarify the impact of individual variants and lead to better interpretation of genotypes and prediction of phenotypes.

This study exemplifies the importance of sharing AST data together with genome data and the potential role for global collaboration such as KlebNET-GSP to utilize these data to enhance the understanding of resistance mechanisms. This is particularly relevant in cases like *bla*_SHV_, where complex evolutionary processes have contributed to the emergence and mobilization of resistant variants within and between the originating species. As the KlebNET-GSP isolate collection grows, we intend to regularly update this analysis to support the growing evidence for *bla*_SHV_ phenotypes, to explore genotype–phenotype variation in the homologous enzymes of other members of the *K. pneumoniae* species complex (*bla*_OKP_ in *K. quasipneumoniae* and *bla*_LEN_ in *K. variicola*) and to undertake similar analyses to inform the understanding of the mechanisms of resistance to other drug classes relevant to the treatment of *K. pneumoniae* infection.

## supplementary material

10.1099/mgen.0.001294 Supplementary Material 1.

10.1099/mgen.0.001294 Supplementary Material 2.

## Abbreviations

AMR: antimicrobial resistance
AST: antimicrobial susceptibility testing
BLI: β-lactamase inhibitor
CLSI: Clinical and Laboratory Standards Institute
ESBL: extended-spectrum β-lactamase
EUCAST: European Committee on Antimicrobial Susceptibility Testing
3GCs: third-generation cephalosporins
MIC: minimum inhibitory concentration
SHV: sulfhydryl variable
