# Supplementary material for: Diversity, functional classification and genotyping of SHV β-lactamases in Klebsiella pneumoniae
Source: Microb Genom. 2024 Oct 21;10(10):001294. doi: 10.1099/mgen.0.001294 (PMC11493186; doi:10.1099/mgen.0.001294)
Supplement: Supplementary Material 2. [file mgen-10-01294-s002.pdf]

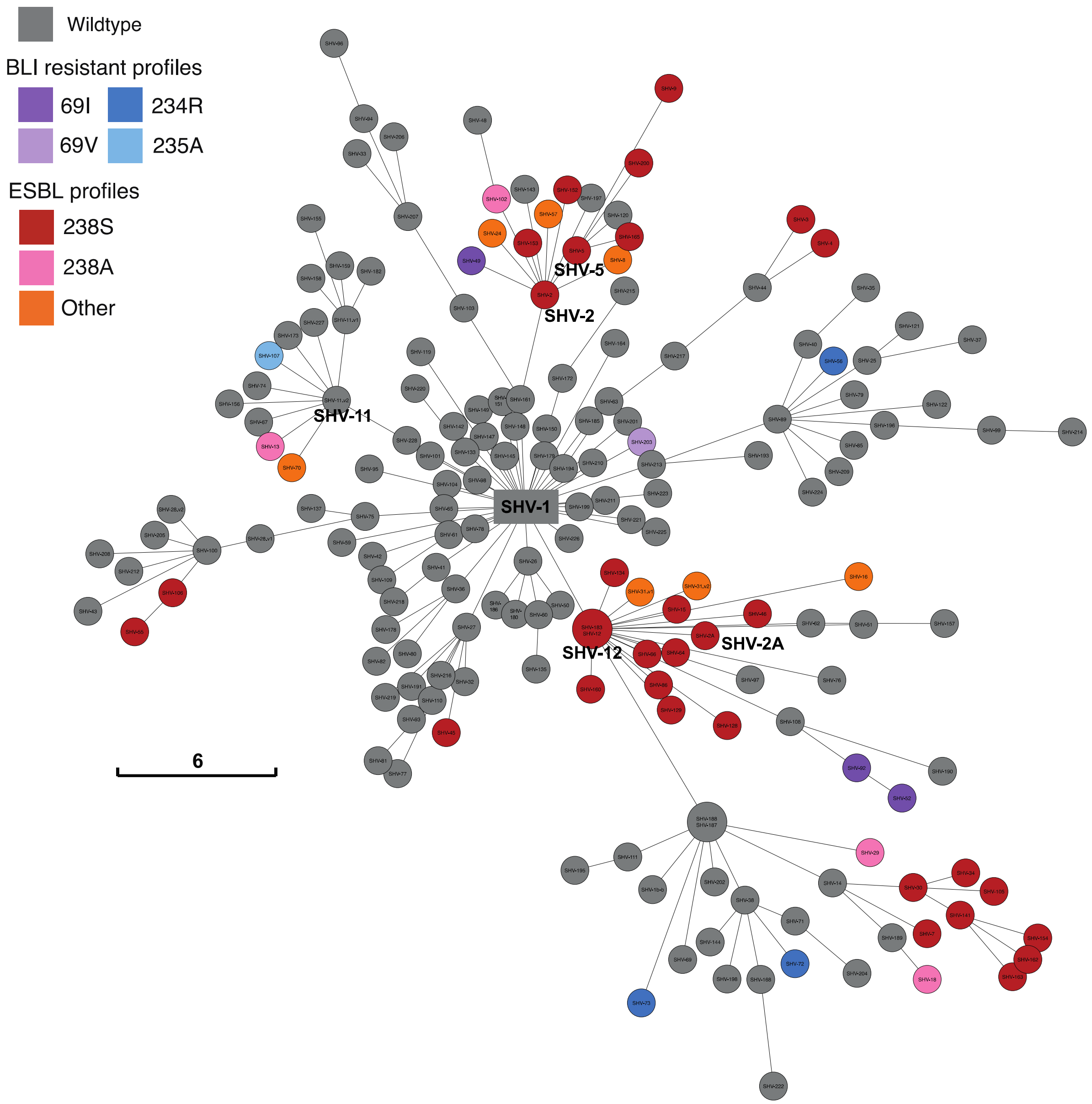

Figure S1: Minimum spanning tree of *bla*SHV alleles.
Minimum spanning tree was inferred using GrapeTree. Size of circles indicates the number of alleles at the same position. Tips are labelled with the SHV allele name, and coloured to indicate the mutation profile (grey=wildtype; red, orange, pink = ESBL mutation profiles; blue, purple = BLI resistant mutation profiles). Scale bar indicates genetic distance.

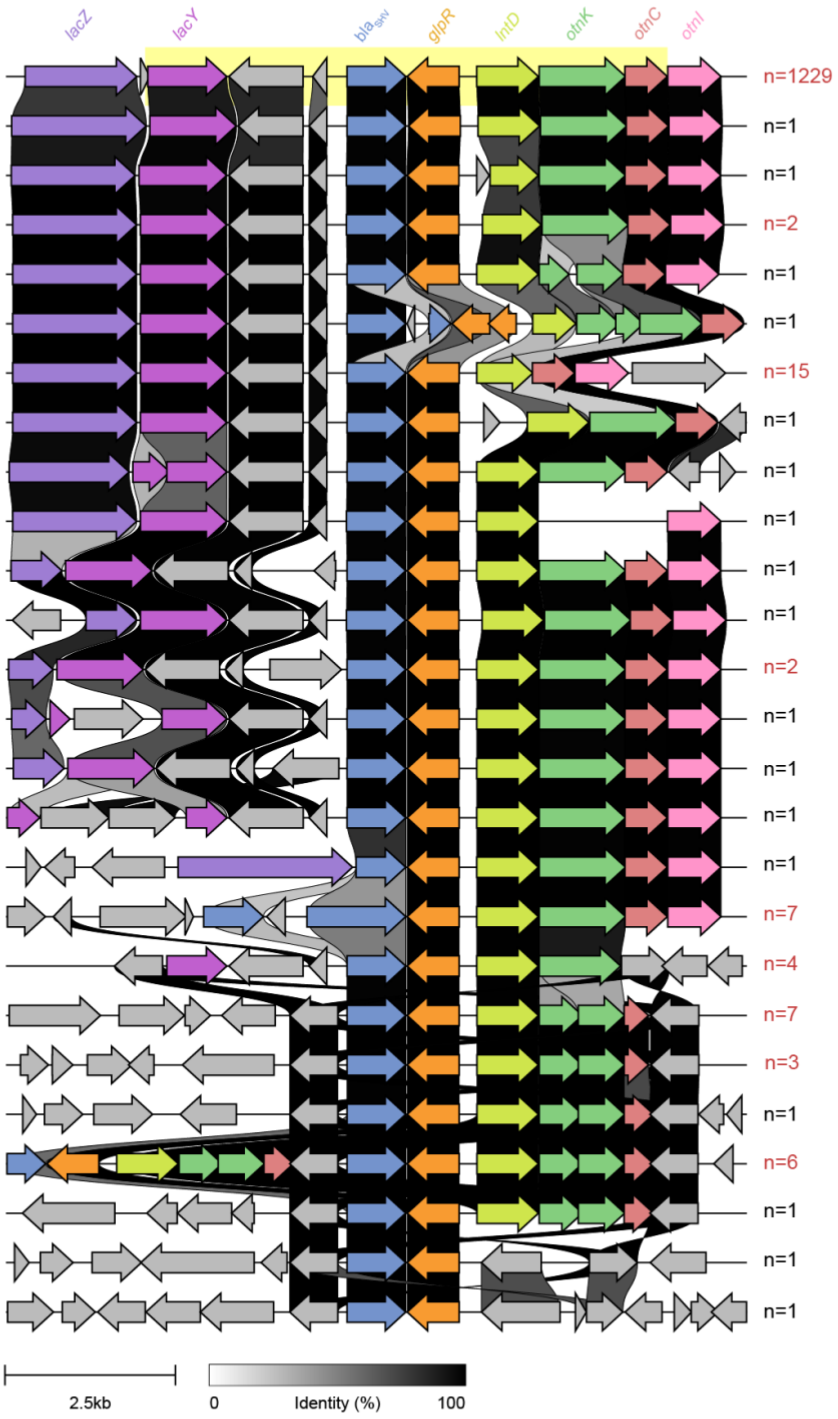

Figure S2: Genetic variation of *bla*$_{SHV}$ flanking regions.
Upstream (5 kbp) and downstream (5 kbp) *bla*$_{SHV}$ were extracted and aligned. The prevalence of each flanking region (≥90% nucleotide sequence similarity) is labelled at the end of each region, where red text indicates flanking regions identified in more than one genome. The 7,585 bp chromosomal SHV collinear block is highlighted in yellow. *Bla*$_{SHV}$ is coloured in blue, while other genes are coloured as labelled. Percent identity between the genes are shown by the gradient scale bar.
